# High-resolution genetic analysis of whole *APC* gene deletions: a report of two cases and patient characteristics

**DOI:** 10.1038/s41439-024-00301-z

**Published:** 2024-12-04

**Authors:** Hiroki Tanabe, Yasuyuki Koshizuka, Kazuyuki Tanaka, Kenji Takahashi, Masami Ijiri, Keitaro Takahashi, Katsuyoshi Ando, Nobuhiro Ueno, Shin Kashima, Takeo Sarashina, Kentaro Moriichi, Kenrokuro Mitsube, Yusuke Mizukami, Mikihiro Fujiya, Yoshio Makita

**Affiliations:** 1 Oncology Center, Asahikawa Medical University Hospital, Asahikawa, Japan; 2 Genetic Oncology Department, Asahikawa Medical University Hospital, Asahikawa, Japan; 3 Department of Genetic Counseling, Asahikawa Medical University Hospital, Asahikawa, Japan; 4 Division of Gastroenterology, Department of Internal Medicine, Asahikawa Medical University, Asahikawa, Japan; 5 Department of Surgery, Asahikawa-Kosei general Hospital, Asahikawa, Japan; 6 Department of Gastroenterology, Asahikawa-Kosei General Hospital, Asahikawa, Japan; 7 Department of Gastroenterology, Japanese Red Cross Asahikawa Hospital, Asahikawa, Japan; 8 Department of Obstetrics and Gynecology, Asahikawa-Kosei General Hospital, Asahikawa, Japan

**Keywords:** Gastrointestinal cancer, Cancer genetics

## Abstract

Familial adenomatous polyposis (FAP) is an autosomal dominant syndrome caused by germline variants in the *APC* gene, leading to the development of numerous colorectal polyps and significantly increases the risk of colorectal cancer. A diagnosis is typically made using colonoscopy, and genetic testing can assist in patient surveillance and carrier identification. Recent advances include the use of whole-genome array comparative genomic hybridization (a-CGH), which provides better resolution of genetic imbalances. We aimed to explore the specific features of FAP patients with whole *APC* gene deletions using high-resolution a-CGH and to compare patient characteristics. Two polyposis patients with whole *APC* deletions were identified, and the lost genetic sizes ranged from 0.3–1.1 Mb. Nervous abnormalities were a characteristic symptom in a patient with a 1.1 Mb loss. A patient with an approximately 0.3 Mb loss, which included the entire *APC* gene, presented a polyposis phenotype without intellectual disability. The comparison of genetic losses, with or without intellectual disability, revealed 7 genetic changes. Consequently, *EPB41L4A* is a candidate gene associated with the neurogenic phenotype.

Familial adenomatous polyposis (FAP), caused by germline variants in the *APC* gene, is an autosomal dominant inherited syndrome. Variant carriers are predisposed to develop hundreds to thousands of colorectal polyps and subsequently develop colorectal cancer. Patients with FAP are typically diagnosed on the basis of the identification of clinical features of colorectal polyposis using colonoscopy. The FAP phenotype is classified as profuse, sparse, or attenuated FAP based on the number of colorectal polyps^1^. Furthermore, patients develop extracolonic features, including fundic gland polyps in the stomach, flat duodenal adenomas, desmoid fibromatosis, adenomas of the ampulla of Vater, hepatoblastoma, and thyroid tumors^2^. The clinical diagnosis is based on the confirmation of colorectal polyposis using colonoscopy. A genetic diagnosis is not required in all FAP cases but is occasionally useful for the surveillance of patients and the diagnosis of carriers. A genotype‒phenotype correlation was reported by Miyaki et al.^1^, and *APC* variations may predict the FAP phenotype of colorectal and extracolonic tumors^3^. *APC* single-gene testing (e.g., sequencing analysis) has been used for molecular genetic testing. Up to 90% of patients with FAP possess *APC* gene variants, whereas the other patients show partial or complete loss of the *APC* gene^4^. Large *APC* deletions have recently been identified using multiplex ligation-dependent probe amplification (MLPA). Chromosomal changes were identified by karyotyping, fluorescence in situ hybridization (FISH), and microarray analysis. Whole-genome array comparative genomic hybridization (a-CGH) is used to identify submicroscopic imbalances, achieving better resolution than conventional karyotypic analysis and providing information on multiple genes^5^.

*APC* is an 8.5 kb cDNA encoding 2843 amino acids and spans approximately 139 kb genetic regions located on chromosome 5q21-q22. Nearly 15,000 variants have been updated on the ClinVar website (https://www.ncbi.nlm.nih.gov/clinvar/?term=APC%5Bgene%5D&redir=gene), in which information on copy number loss and insertion/deletion polymorphisms is also deposited. *APC* deletions occur as large, cytogenetically visible rearrangements, and a recent literature review described 5q deletion syndrome with dysmorphic features, behavioral disturbances, and intellectual disability^6,7^. In this study, we performed high-resolution a-CGH analysis to identify deleted regions on chromosome 5q. We report 2 cases with whole *APC* deletions to elucidate the differences in patient characteristics between the two patients.

The patients were diagnosed with advanced colorectal cancer with polyposis by colonoscopy (Table). The patients underwent total resection of the large intestine and received chemotherapy for metastatic cancer, but Patient 1 could not continue to receive later lines of chemotherapy. Patient 2 received continuous chemotherapy until disease progression. Patient 1 had mild intellectual disability without any physical abnormalities or developmental delays. Patient 2 presented with multiple fundic gland polyps and duodenal adenoma via esophagogastroduodenoscopy. Patient 1 did not undergo endoscopy because of her poor understanding.

These patients were subjected to comprehensive genomic panel (CGP) analysis to identify suitable chemotherapies for metastatic advanced colorectal cancers. These were negative for genetic variants in the genomic panel (OncoGuide^TM^ NCC oncopanel, Sysmex, Hyogo, Japan) and were therefore subjected to further genetic analyses. We did not detect an *APC* variant in these cases; however, complete *APC* deletion was suspected on the basis of the observation of a low amplicon depth in the long arm of chromosome 5 (Supplementary Fig. S1). The retrospective clinical study protocol was approved by Asahikawa Medical University ethical committee (#24075).

**Fig. 1:**
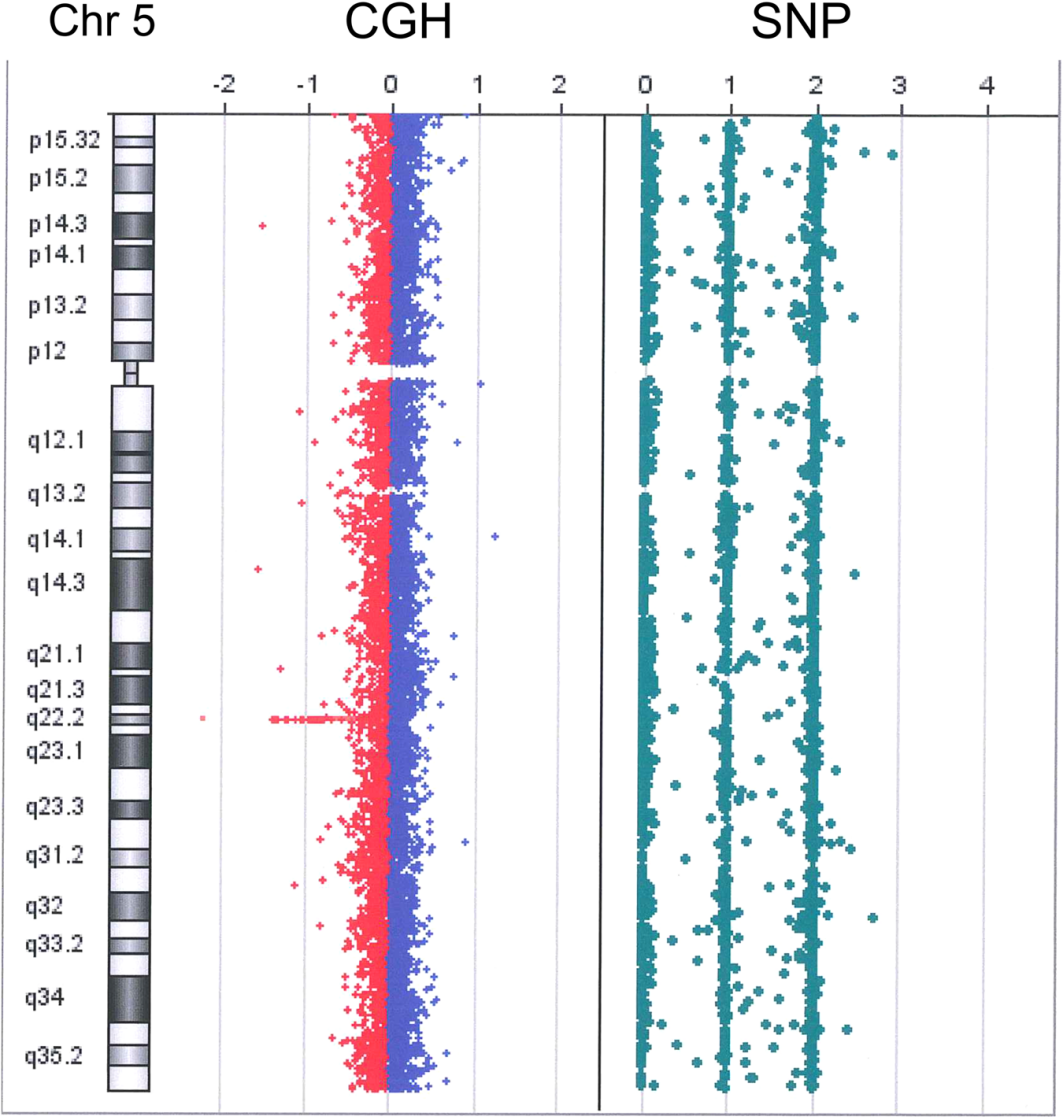
Comparative genomic hybridization analysis of chromosome 5. A heterozygous deletion of 5q22 was detected in Patient 2.

MLPA analysis of genomic DNA was performed using the SALSA MLPA probe mix P043 APC and a reagent kit (MRC-Holland, Amsterdam, Netherlands). The Coffalyser Net software program (v.220513.1739) was used for the data analysis. All the MLPA probe signals were reduced by half relative to those of the controls (Supplementary Fig. S2), indicating that the whole *APC* gene was deleted. To identify the deleted regions, genome-wide a-CGH was performed (Fig. 1). Data analysis was performed using GenetiSure Dx Postnatal Analysis Method v.1 (Agilent Technology, Tokyo, Japan). Large deletions on chromosome 5q were observed in patients 1 and 2 (Table 1, Fig. 2), and the sizes (minimum-maximum) of the deletions were 1.07–1.12 Mb and 0.25–0.33 Mb, respectively. Decipher (DatabasE of Chromosomal Imbalances and Phenotype via Ensemble Resources; https://www.deciphergenomics.org) was accessed and used to interpret the results.

**Fig. 2:**
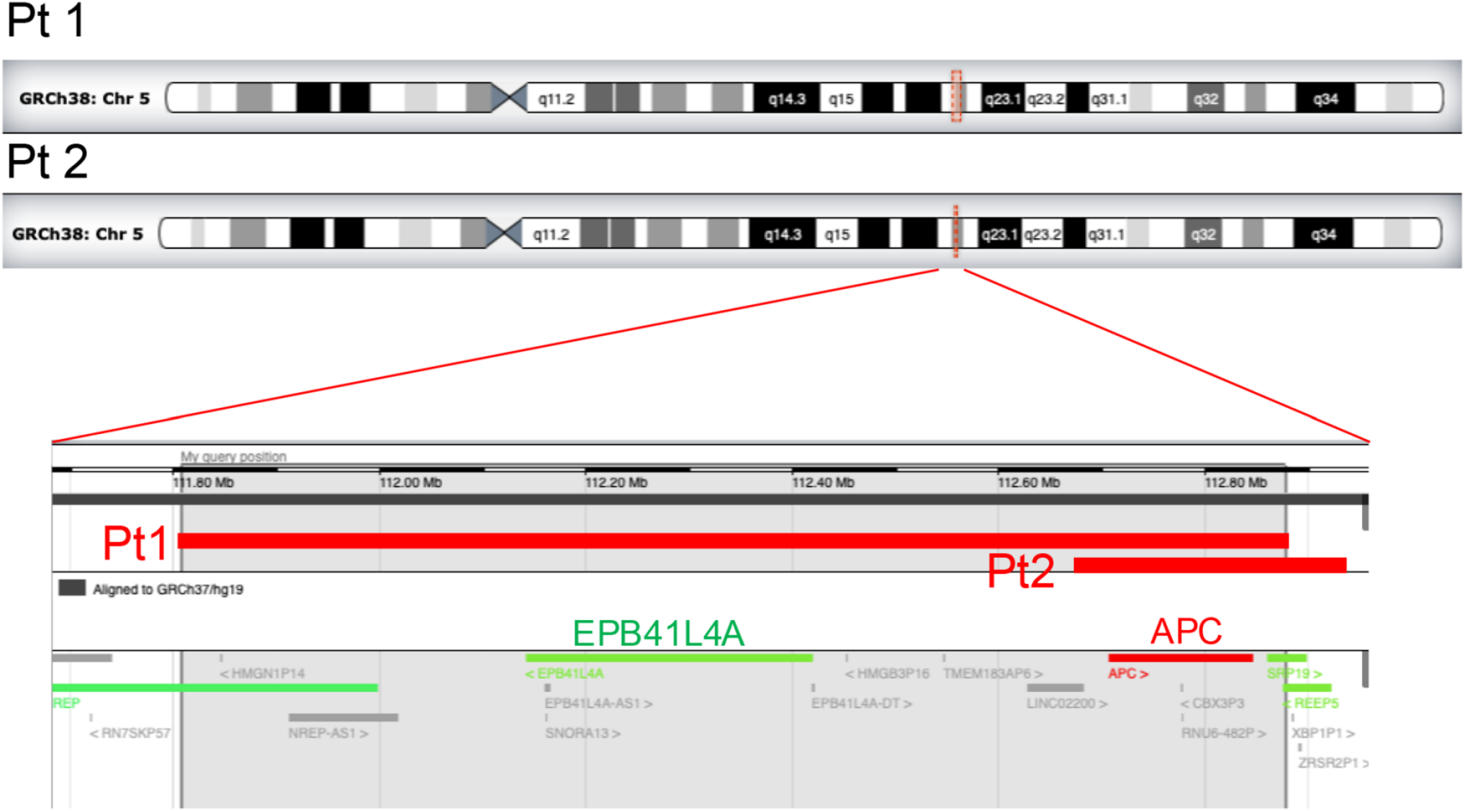
Comparative genomic hybridization profiles obtained from the 2 patients. The deleted areas are indicated with red squares in the chromosome 5 ideograms. An enlargement of the deleted lesions of Patient 2 is shown in the lower panel, with the deletions highlighted by the red bars in the 2 patients (Pt 1 and Pt 2). Several genes near the *APC* locus are included in this region.

**Table 1:** Patient characteristics and annotation of array comparative genomic hybridization data.

Patient	Age	Sex	Nervous system	Growth abnormality	Stomach	Duodenum	Neoplasm	Location (GRCh37)	GRCh38	Size	Annotation
1	27 y	Female	intellectual disability	none	not tested	not tested	d-colon cancer	5:111143360-112213143	5:111807663-112877446	1.07–1.12 Mb	NREP, NREP-AS1, EPB41L4A-AS1, SNORA13, LOC101927023, EPB41L4A-DT, LIMC02200, LOC102467216, APC, SRP19, REEP5
2	31 y	Female	none	none	fundic gland polyps	deodenal adenoma	s-colon cancer	5:112015411-112269758	5:112679714-112934061	0.25–0.33 Mb	LOC102467216, APC, SRP19, REEP5

*APC* is a tumor suppressor gene responsible for the production of adenomatous polyposis coli (APC), which regulates β-catenin and plays a crucial role in cell communication, signaling, and growth. *APC* variants are inherited in an autosomal dominant manner. Approximately 75–80% of patients with *APC*-associated polyposis have an affected parent^8^. The *APC* gene was analyzed via Sanger sequencing, next-generation sequencing, and long-read sequencing. For conventional clinical use, the target sequence is chosen primarily as a gene panel, and MLPA is used in a complementary manner^9^. These *APC*-targeted genetic tests fail to identify cases with whole genetic deletions in chromosome 5. MLPA is recommended for patients without genetic variation because it can detect genomic deletions and duplications. However, information on areas deleted from the primer sets may be lost. Chromosomal analysis techniques have advanced, with karyotyping, fluorescence in situ hybridization, and a-CGH now being commercially available^10^. A-CGH is utilized for the genetic testing of individuals with developmental delay, intellectual disability, autism spectrum disorder, or multiple congenital anomalies. The International Standard Cytogenomic Array Consortium recommended the use of a chromosomal microarray for these patients^11^. In our experiment, the a-CGH assay revealed the regions involving the candidate genes associated with intellectual disability. Patient 1 experienced intellectual disability, whereas Patient 2 did not. The differences between the 2 patients included the following 6 candidate genes: *NREP, NREP-AS1, EPB41L4A, EPB41L4A-AS1, SNORA13, and EPB41L4A-DT*. With respect to neurological disorders, *EPB41L4A* has been reported in previous studies and a literature review. Extensive exploration of disease genes in neurological disorders has been performed via whole-exome sequencing, and 33 genes that have not been previously reported were identified^11^. *EPB41L4A* was included among the candidate genes. This gene was recently reported to be a candidate gene contributing to neurological disorders in patients with 5q interstitial deletions^12^. This review provides novel clinical and molecular information for the management of patients with 5q deletions that involve *APC*. This paper mainly discusses the severity of intellectual disability in these cases and does not address polyposis^13^. *KCNN2* was hypothesized to be the most likely candidate gene contributing to intellectual disability. However, *KCNN2* was not included among the 6 candidate genes.

*EPB41L4A* encodes the 41L4A erythrocyte membrane protein band 4.1 like A, which is a member of the band 4.1 protein superfamily. Members of this superfamily are thought to play important roles in regulating interactions between the cytoskeleton and plasma membrane and contain an amino-terminal conserved domain that binds glycophorin C. This gene product is thought to be involved in the beta-catenin signaling pathway^14^. *EPB41L4A* expression was significantly reduced when beta-catenin was depleted in SW480 cells, a human cancer cell line that constitutively accumulates β-catenin. These results support the view that *EPB41L4A* is an important component of the β-catenin/Tcf pathway and is likely related to the determination of cell polarity or proliferation^15^. *EPB41L4A* is a downstream transcriptional target of β-catenin/Tcf41 and has been linked to colon cancer. This clinical evidence was shown by a multidimensional genomic analysis employing chromosomal microarray profiling, optical mapping, long-read genome sequencing, and RNA sequencing combined with FISH and standard PCR of genomic and complementary DNA. Constitutional chromothripsis of the *APC* locus was identified as the cause of the genetic predisposition to colorectal cancer in a patient with FAP in whom *APC* variants were not detected by next-generation sequencing panel analysis of *APC* and other polyposis-associated genes^16^.

Genetic analysis of *APC* variants in patients with FAP has been performed worldwide. The strategy for the molecular diagnosis of FAP involves full sequencing analysis followed by screening for copy number variants via a CGH or MLPA^17^. The resolution of genetic assays depends on the size and interval of the designed probes. The resolution of a-CGH is improving, and the current mean resolution of the commercially available platform is 150 kb^18^. The length of the deletion in Patient 2 was approximately 300 kb, and the minimum length deposited in Decipher (Patient 3371809) was 260 kb. A case series of *APC* rearrangement in FAP revealed 3 patients with partial or whole *APC* deletions of 30, 265, and 921 kb in length^19^. The *APC* gene is 139 kb in length, and whole-APC deletion can be detected by the Agilent Technologies platform, as in our 2 cases. Patient 1 showed a 1.1 Mb deletion, which included 11 genes, and a comparative study with Patient 2 was useful for identifying the candidate gene associated with intellectual disability. In the 3 previous patients, *APC* was deleted, whereas *EPB41L4A* was preserved. No information on intelligence is shown in the report. Yamaguchi Y et al. reported a case involving a deletion on chromosome 5, namely 5q22.1-22.2. This deletion was associated with polyposis, but notably, it was not associated with intellectual disability^20^. The deletion, identified via MLPA and FISH, was approximately 1.7 Mb in size and included two genes: *APC* and *EPB41L4A*. Although this deletion is located close to the smaller 1.1 Mb deletion observed in Patient 1 of this study, the specific symptoms affecting the nervous system differed between the cases.

In a broader study of cases stored in Decipher (Supplementary Fig. S3), other patients with deletions affecting the *EPB41L4A* gene were analyzed using a technique called microarray. Many, though not all, of these patients had intellectual disabilities. The probability of haploinsufficiency (pHaplo) for *EPB41L4A* is 0.25, indicating a relatively low likelihood of symptoms occurring due to a single copy loss of this gene^13^. Thus, the risk that a loss-of-function mutation in this gene would lead to intellectual disability is considered low. However, 10 out of 15 patients with loss of heterozygosity in the *EPB41L4A* gene had intellectual disability (Supplementary Table S1). *EPB41L4A* may serve as a potential predictor gene of intellectual disability, although its effect may exhibit incomplete penetrance. To draw more definitive conclusions about the link between this genetic deletion and phenotypes, more case data are needed for comparison and analysis. A-CGH is a powerful tool for revealing genetic imbalances.

In conclusion, a-CGH can detect whole-*APC* deletions and enable the identification of deleted lesions and genes, providing more comprehensive information compared to the identification of single *APC* genetic losses. Our analysis of the deposited cases with whole-*APC* deletions indicated that intellectual disability is one of the major symptoms. We identified *EPB41L4A* as a likely candidate gene contributing to neurogenic symptoms.

## Note added to proof

The authors declare that Patient 1 was previously described in a data report^21^.

## HGV Database

The relevant data from this Data Report are hosted at the Human Genome Variation Database at 10.6084/m9.figshare.hgv.3456, 10.6084/m9.figshare.hgv.3459.

## Supplementary information

Supplementary file

## References

[1] Miyaki, M. et al. Difference in characteristics of APC mutations between colonic and extracolonic tumors of FAP patients: variations with phenotype. *Int. J. Cancer* **122**, 2491–2497 (2008). 18224684 10.1002/ijc.23390

[2] Quadri, M. et al. APC rearrangements in familial adenomatous polyposis: heterogeneity of deletion lengths and breakpoint sequences underlies similar phenotypes. *Fam. Cancer* **14**, 41–49 (2015). 25159889 10.1007/s10689-014-9750-3

[3] Nallamilli, B. R. R. & Hegde, M. Detecting APC gene mutations in familial adenomatous polyposis (FAP). *Curr. Protoc. Hum. Genet.* **92**, 10.8.1–10.8.16 (2017). 28075483 10.1002/cphg.29

[4] Lagarde, A. et al. Germline APC mutation spectrum derived from 863 genomic variations identified through a 15-year medical genetics service to French patients with FAP. *J. Med. Genet.* **47**, 721–722 (2010). 20685668 10.1136/jmg.2010.078964

[5] Michils, G. et al. Large deletions of the APC gene in 15% of mutation-negative patients with classical polyposis (FAP): a Belgian study. *Hum. Mutat.* **25**, 125–134 (2005). 15643602 10.1002/humu.20122

[6] Miller, D. T. et al. Consensus statement: chromosomal microarray is a first-tier clinical diagnostic test for individuals with developmental disabilities or congenital anomalies. *Am. J. Hum. Genet.* **86**, 749–764 (2010). 20466091 10.1016/j.ajhg.2010.04.006 PMC2869000

[7] Casper, M. et al. Multidisciplinary treatment of desmoid tumours in Gardner’s syndrome due to a large interstitial deletion of chromosome 5q. *QJM* **107**, 521–527 (2014). 24554300 10.1093/qjmed/hcu036

[8] Wallis, Y. L., Morton, D. G., McKeown, C. M. & Macdonald, F. Molecular analysis of the APC gene in 205 families: extended genotype-phenotype correlations in FAP and evidence for the role of APC amino acid changes in colorectal cancer predisposition. *J. Med. Genet.* **36**, 14–20 (1999). 9950360 PMC1762945

[9] Nielsen, M. et al. Genotype-phenotype correlations in 19 Dutch cases with APC gene deletions and a literature review. *Eur. J. Hum. Genet.* **15**, 1034–1042 (2007). 17568392 10.1038/sj.ejhg.5201871

[10] Castellsagué, E. et al. Detection of APC gene deletions using quantitative multiplex PCR of short fluorescent fragments. *Clin. Chem.* **54**, 1132–1140 (2008). 18487285 10.1373/clinchem.2007.101006

[11] Waggoner, D. ACMG Professional Practice and Guidelines Committee et al. Yield of additional genetic testing after chromosomal microarray for diagnosis of neurodevelopmental disability and congenital anomalies: a clinical practice resource of the American College of Medical Genetics and Genomics (ACMG). *Genet. Med.* **20**, 1105–1113 (2018). 29915380 10.1038/s41436-018-0040-6 PMC6410698

[12] Alazami, A. M. et al. Accelerating novel candidate gene discovery in neurogenetic disorders via whole-exome sequencing of prescreened multiplex consanguineous families. *Cell Rep.* **10**, 148–161 (2015). 25558065 10.1016/j.celrep.2014.12.015

[13] Privitera, F. et al. APC-related phenotypes and intellectual disability in 5q interstitial deletions: a new case and review of the literature. *Genes* **14**, 1505 (2023). 37510409 10.3390/genes14071505 PMC10379344

[14] Yang, Fan & Lv, Shixu LncRNA EPB41L4A-AS1 regulates cell proliferation, apoptosis and metastasis in breast cancer. *Ann. Clin. Lab. Sci.* **52**, 3–11 (2022). 35181612

[15] Bin, J. et al. Long noncoding RNA EPB41L4A-AS1 functions as an oncogene by regulating the Rho/ROCK pathway in colorectal cancer. *J. Cell Physiol.* **236**, 523–535 (2021). 32557646 10.1002/jcp.29880

[16] Scharf, F. et al. Constitutional chromothripsis of the APC locus as a cause of genetic predisposition to colon cancer. *J. Med. Genet.* **59**, 976–983 (2022). 34911816 10.1136/jmedgenet-2021-108147 PMC9554066

[17] Meuller, J. et al. Identification of genomic deletions of the APC gene in familial adenomatous polyposis by two independent quantitative techniques. *Genet. Test.* **8**, 248–256 (2004). 15727247 10.1089/gte.2004.8.248

[18] Manning, M. & Hudgins, L. Professional Practice and Guidelines Committee Array-based technology and recommendations for utilization in medical genetics practice for detection of chromosomal abnormalities. *Genet. Med.* **12**, 742–745 (2010). 20962661 10.1097/GIM.0b013e3181f8baad PMC3111046

[19] Sieber, O. M. et al. Whole-gene APC deletions cause classical familial adenomatous polyposis, but not attenuated polyposis or “multiple” colorectal adenomas. *Proc. Natl Acad. Sci. USA* **99**, 2954–2958 (2002). 11867715 10.1073/pnas.042699199 PMC122454

[20] Yamaguchi, T. et al. A large deletion of chromosome 5q22.1-22.2 associated with sparse type of familial adenomatous polyposis: report of a case. *JPN J. Clin. Oncol.* **44**, 1243–1247 (2014). 25324480 10.1093/jjco/hyu150

[21] Tanabe, H. et al. Genomic insights into familial adenomatous polyposis: unraveling a rare case with whole APC gene deletion and intellectual disability. *Hum. Genome Var.* **11**, 13 (2024). 38548799 10.1038/s41439-024-00270-3 PMC10978947

