# Supplementary material for: High-resolution genetic analysis of whole APC gene deletions: a report of two cases and patient characteristics
Source: Hum Genome Var. 2024 Dec 4;11:46. doi: 10.1038/s41439-024-00301-z (PMC11618449; doi:10.1038/s41439-024-00301-z)
Supplement: Supplementary file 1 — Supplementary file [file 41439_2024_301_MOESM1_ESM.pdf]

Supplementary Figure S1

Results of the cancer genomic panel analysis. The depth of the *APC* gene was low in the tumor and blood samples.

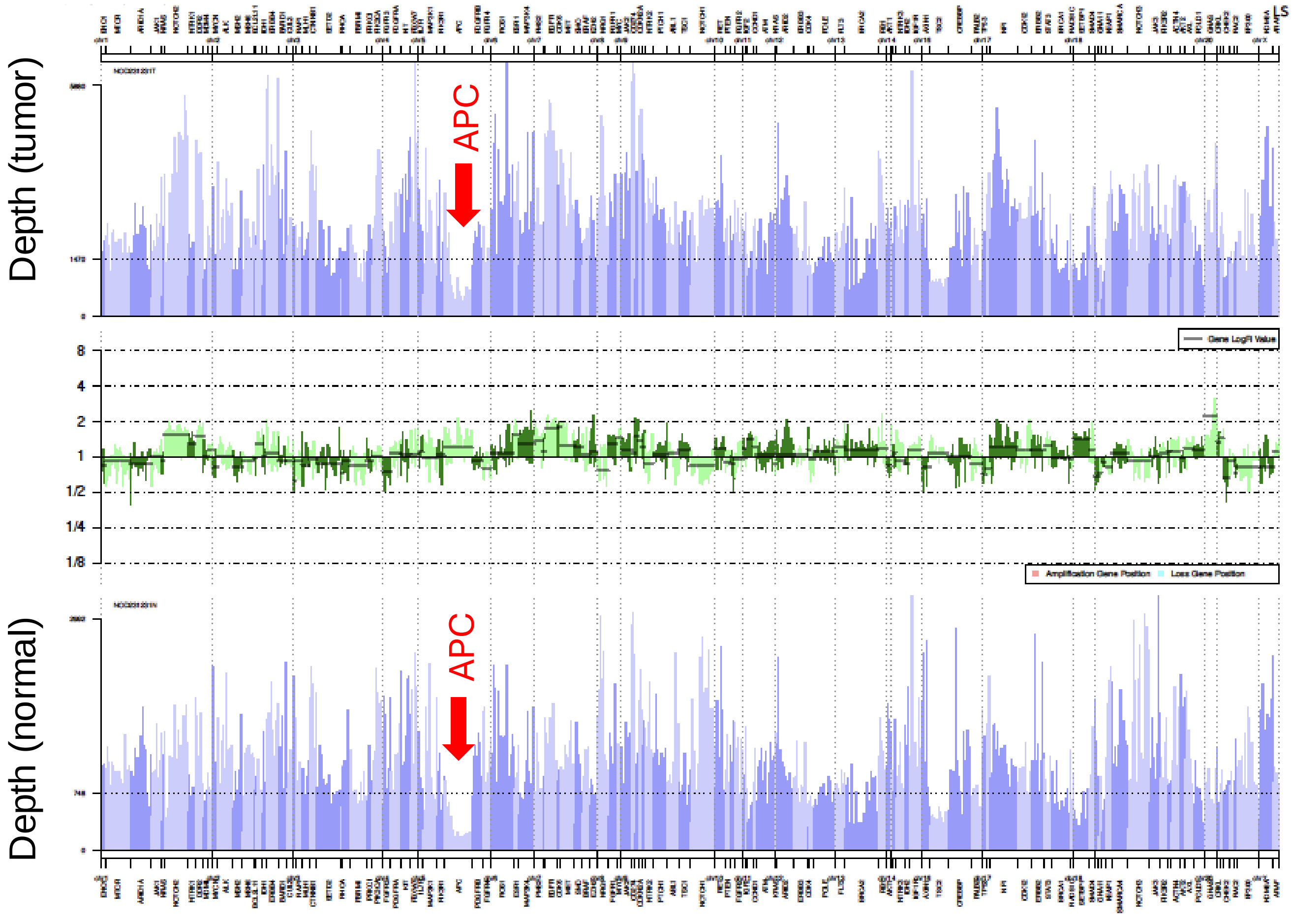

Supplementary Figure S2

Multiplex ligation-dependent probe amplification assay (MLPA). MLPA detected a heterozygous deletion of the entire *APC* gene, with red dots indicating a depth ratio of 0.5.

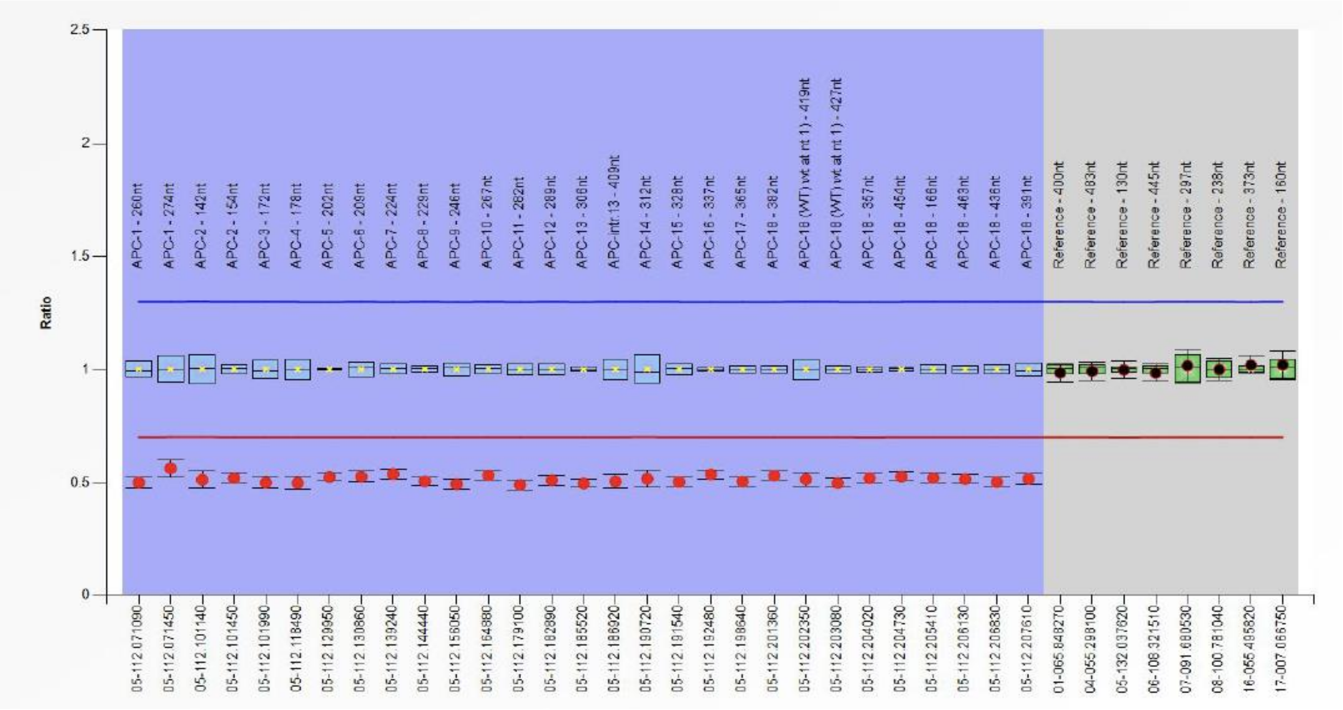

Supplementary Figure S3

Chromosome 5 ideogram and the patients with whole *EPB41L4A* deletion. Decipher (DatabaseE of Chromosomal Imbalances and Phenotype using Ensemble Resources-https://www.deciphergenomics.org) was accessed and used in the interpretation of the results. Concerning database analysis, the clinical information of the cases with whole *EPB41L4A* deletions were extracted from Decipher using lesion 5:112141829-112419935 (CRCh38). Fifteen cases with pathogenic and likely pathogenic copy number loss variants were extracted from the database.

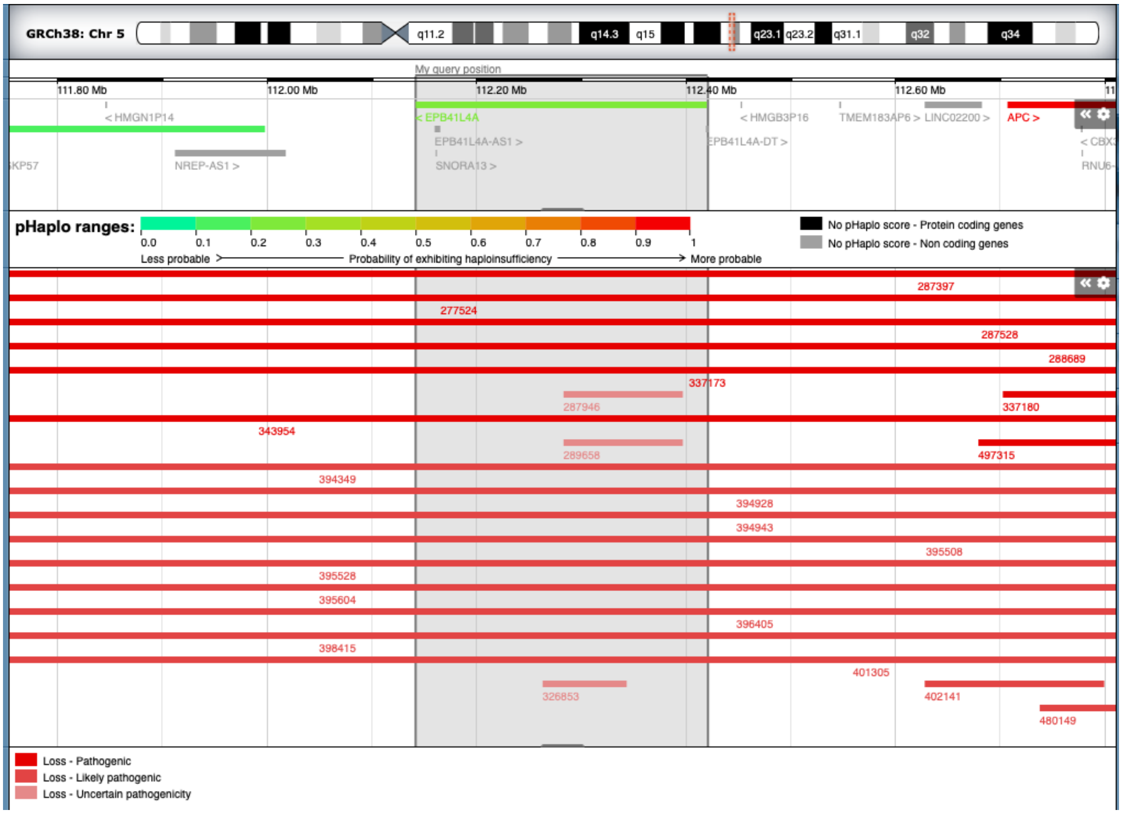

Supplementary Table S1

Characteristics of the patients with *EPB41L4A* copy number loss in Decipher

EPB41L4A	patient	age	sex	location	size	abnormality of nervous system
pathogenic	287397	11y	46XX	5:109453243-118380159	8.93Mb	intellectual disability
	277524	ND	46XY	5:110053938-119564382	9.51Mb	
	287528	3y	46XY	5:109514099-116638060	7.12Mb	intellectual disability
	288689	ND	unknown	5:102185046-119585830	17.40Mb	delayed fine motor development, developmental delay
	337173	24w	46XY	5:108178781-125671740	17.49Mb	
	343954	ND	46XY	5:99318010-120664305	21.35Mb	global developmental delay
likely pathogenic	394349	42y	46XY	5:93038538-115936404	22.90Mb	intellectual disability
	394928	25y	46XY	5:110336400-128636408	18.30Mb	intellectual disability
	394943	54y	46XX	5:110336400-128636408	18.30Mb	intellectual disability
	395508	38y	46XX	5:105236400-122136406	16.90Mb	
	395528	31y	46XY	5:93038538-115936404	22.90Mb	intellectual disability
	395604	less than 1y	46XX	5:93038538-115936404	22.90Mb	intellectual disability, autistic behavior
	396405	34y	46XX	5:110336400-128636408	18.30Mb	intellectual disability, abnormal central sensory function, delayed speech and language development
	398415	39y	46XY	5:93038538-115936404	22.90Mb	intellectual disability
	401305	1y	46XX	5:104110212-120252670	16.14Mb	intellectual disability

ND: not described
